# Supplementary material for: Patient complexity assessment tools containing inquiry domains important for Indigenous patient care: A scoping review
Source: PLoS One. 2022 Aug 31;17(8):e0273841. doi: 10.1371/journal.pone.0273841 (PMC9432764; doi:10.1371/journal.pone.0273841)
Supplement: Attachment — Submitted filename: PatientComplexityReview-v3.pdf [file pone.0273841.s002.pdf]

PLOS: Patient complexity assessment tools containing inquiry domains important for Indigenous patient care: A scoping review.

MANUSCRIPT REVIEW

Summary / General Commentary

Concerning the content of the review, no major errors were apparent. Under close scrutiny, the inquiry was both factually accurate and offered a balanced, skilfully coherent interpretation of prior art. Areas for improvement are relatively inconsequential, rarely warranting more than a few additional lines of analysis or a graphic to deepen the interpretation of findings.

Positive Remarks

1. This review achieved an impressive balance between technicality and understandability, allowing it to remain **informative and non-obvious** to future readers with a wide range of expertise.

2. All data and discussions were communicated firmly. Explanations of the circumstances experienced by indigenous populations are **well-developed and informed, never compromising on accuracy or tact.**

3. Among many others, the *"Additional Considerations"* section (particularly the connections that it drew to deficit-centric narratives) was sharp, insightful, and diverse - **demonstrating a commendable level of nuance and depth of exploration on the part of the authors.**

Recommendations

Domains of Inquiry

The introduction of your study suggests that all PCATs were primarily assessed on their agreement with the facets of E4E.

Furthermore, lines 75-76 state that the E4E framework included *"social and economic resource disparities, and the accumulation of adverse life experiences,"* Given that these factors are the basis for much of the analysis that will be conducted in the review, they should be covered in more depth and in their entirety from the outset.

Additionally, the process by which it is decided that an item in a given PCAT aligns with E4E or not is not immediately clear. Along with the mention of the expert group that evaluated the validity of each category, **this can be supplemented with hypothetical examples or related devices:**

"Patient to self-report if they are religious" - Example of tangential agreement with "culture as therapeutic" requirement because regular practice of religion is not captured.

vs.

"Patient to self-report less than weekly participation at religious services" - Example of full agreement with "culture as therapeutic" requirement because regular practice is captured.

Such examples would better illustrate why certain items in a PCAT would be judged as agreeing, disagreeing, or partially agreeing to the fundamental aspects of E4E, and would make the panel feel less like a black box.

Highlighting the Influence of Recent PCATs

As outlined in table 1, it appears that by reiterating Marcoux's protocol in the present day, nine additional complexity assessment tools were identified.

These PCATs must be further emphasized, since the value proposition of this review hinges (to a large degree) on how these new instruments affect the original conclusion drawn by Marcoux et al.

More specifically, they provide important context into PCATs that simply did not exist when Marcoux published her research in 2017, and could realistically be used to delineate a continued failure to consider indigenous social realities in the past half-decade.

Whether the domains of inquiry in these new instruments seem to support Marcoux's original conclusion or oppose it in some respects, the changes they warrant to the existing consensus should be more prominent in the *"Discussion"* or *"Conclusion"*.

Methodology Rationale

Little rationale was provided for why the current review followed the same methodology as that of Marcoux et al.

Although it is clear among indigenous health researchers who have read that Marcoux's methodology was reliable and exhaustive, this is not necessarily a given for readers outside the field. All considered, this could be resolved quite directly with some **additional commentary in the Protocol section that explains why the same protocol was continued.**

Synthesizing Bird's-Eye Trends

Might there be domains of inquiry that are consistently underrepresented across many of the PCATs that were analyzed? Although this is a scoping review, gaining a more numerical understanding of exactly which dimensions are lacking will lead to a more definitive response and render the conclusion more precise.

More emphasis should be placed on inter-test trends - both to make your inquiry more enriching, and to better meet your stated aim of assessing the want for a dedicated PCAT for indigenous populations.

In the case of your review, **an extra graph that summarizes the cumulative exposure each E4E factor cumulatively receives from all the PCATs** would contribute greatly to the tangibility of the findings, beyond the general claim that indigenous social realities are lacking.

Making Conclusions More Comparative

Stating that MCAM, OCCAM, and other PCATs largely neglect indigenous social realities is a good start. However, this leaves gaps for further questioning, since a small (but non-trivial) portion of the community still believes that PCATs perform poorly in general - whether the patient is indigenous or not.

To negate any such counterpoint, it would be useful to compare the alignment of each instrument with both indigenous and non-indigenous populations. Your review makes a more substantial claim: **not only do current PCATs neglect domains relevant to indigenous patients; they do so while being relatively inclusive of non-indigenous patients. In this way, you can highlight a disparity.**

But to do this, this part of your analysis must be two-sided. This does not call for any major reworking, **save for a few more lines in either the discussion or conclusion to make it clear that there is evidence of one group being neglected in these assessments.**

At the very least, it would emphasize that a considerable amount of forethought was directed into this inquiry.

Miscellaneous notes

In the OCCAM section of table 3 (pg. 15) it appears that the word “of” is missing in “This item partially addresses adverse life experiences that shape health”.

Final Remarks

It has been some time since I last reviewed a paper in this specific niche. This was a welcome and refreshing piece that I genuinely look forward to reading following publication, and whose potential for social good and reconciliation is especially compelling.
